# Identification and Validation of T-Cell Exhaustion Signature for Predicting Prognosis and Immune Response in Pancreatic Cancer by Integrated Analysis of Single-Cell and Bulk RNA Sequencing Data

**DOI:** 10.3390/diagnostics14060667

**Published:** 2024-03-21

**Authors:** Yaowu Zhu, Li Tan, Danju Luo, Xiong Wang

**Affiliations:** 1 Department of Laboratory Medicine, Tongji Hospital, Tongji Medical College, Huazhong University of Science and Technology, Wuhan 430030, China; yaowu_zhu@163.com; 2 Department of Infection Control, Tongji Hospital, Tongji Medical College, Huazhong University of Science and Technology, Wuhan 430030, China; tanlidyx@126.com; 3 Department of Pathology, Union Hospital, Tongji Medical College, Huazhong University of Science and Technology, Wuhan 430030, China

**Keywords:** pancreatic cancer, T-cell exhaustion, immunotherapy, risk model, SPOCK2

## Abstract

Purpose: Pancreatic cancer (PACA) is one of the most fatal malignancies worldwide. Immunotherapy is largely ineffective in patients with PACA. T-cell exhaustion contributes to immunotherapy resistance. We investigated the prognostic potential of T-cell exhaustion-related genes (TEXGs). Methods: A single-cell RNA (scRNA) sequencing dataset from Tumor Immune Single-Cell Hub (TISCH) and bulk sequencing datasets from the Cancer Genome Atlas (TCGA) and Genotype-Tissue Expression (GTEx) were used to screen differentially expressed TEXGs. Kaplan–Meier survival, LASSO regression, and univariate/multivariate Cox regression analyses were performed to construct a TEXG risk model. This model was used to predict the prognosis, tumor immune microenvironment, and immunotherapy response. The PACA cohorts from the ICGC and GSE71729 datasets were used to validate the risk model. Pan-cancer expression of SPOCK2 was determined using the TISCH database. Results: A six-gene (*SPOCK2*, *MT1X*, *LIPH*, *RARRES3*, *EMP1*, and *MEG3*) risk model was constructed. Patients with low risk had prolonged survival times in both the training (TCGA-PAAD, *n* = 178) and validation (ICGC-PACA-CA, ICGC-PAAD-US, and GSE71729, *n* = 412) datasets. Multivariate Cox regression analysis demonstrated that the risk score was an independent prognostic variable for PACA. High-risk patients correlated with their immunosuppressive status. Immunohistochemical staining confirmed the changes in TEXGs in clinical samples. Moreover, pan-cancer scRNA sequencing datasets from TISCH analysis indicated that SPOCK2 may be a novel marker of exhausted CD8^+^ T-cells. Conclusion: We established and validated a T-cell exhaustion-related prognostic signature for patients with PACA. Moreover, our study suggests that SPOCK2 is a novel marker of exhausted CD8+ T cells.

## 1. Introduction

Pancreatic cancer (PACA) is the seventh leading cause of cancer-related death, with slightly increased incidence and mortality rates in many countries [1]. Moreover, PACA is expected to surpass breast cancer and become the third leading cause of cancer-related death by 2025 [2]. The prognosis of PACA is poor, with a 5-year survival rate of approximately 2–9% [3]. In most cases, conventional therapies, including surgical interventions, chemotherapy, radiotherapy, and targeted therapies, are insufficient [4,5]. Immunotherapy has advanced remarkably for many malignant tumors, and PACA is refractory to almost all United States Food and Drug Administration-approved immunotherapies. PACA exhibits a “cold” tumor immune microenvironment (TIME) characterized by poor T-cell infiltration and low mutational burden. Strategies that combine chemotherapy with immune checkpoint blockade (ICB) and dual checkpoint blockade exhibited minimal activity against PACA [6]. However, personalized immunotherapy based on the PACA genotype and immunological heterogeneity may provide novel insights [7,8].

T-cell exhaustion (TEX) is one of the main causes of immunotherapy failure and is characterized by sustained expression of inhibitory receptors, poor effector function, and a distinct transcriptional state, epigenome, and metabolic profile different from that of functional effector or memory T-cells [9,10]. TEX limited the effector function and activity of T-cells during chronic antigen stimulation and limits the response against cancer, leading to disease progression [10]. Furthermore, CD8^+^ TEX (CD8TEX) is driven by massive immunosuppressive signals including nutrient deficiency, hypoxia, proinflammatory cytokines, and chronic T-cell receptor signaling in the tumor microenvironment (TME), which is regarded as the main responder to ICB, and is correlated with poor prognosis in numerous cancers [11]. Exhausted CD8^+^ T-cells express low levels of CD122 and CD127, leading to poor response to interleukin (IL)-7- and IL-15-mediated homeostatic self-renewal [9,12]. Moreover, several inhibitory receptors coregulate TEX, including programmed cell death protein 1 (PD-1), lymphocyte activation gene 3 (LAG3), CD160, CD244, B- and T-lymphocyte attenuator, cytotoxic T-lymphocyte-associated protein 4 (CTLA4), and T-cell immunoglobulin and mucin domain-containing protein 3 (TIM3) [9,13]. Transcriptional studies revealed that the transcriptional repressor Blimp-1 was enriched in exhausted CD8^+^ T-cells and correlated with the upregulation of inhibitory receptors [14]. In addition, immunological markers of senescence were downregulated in exhausted CD8^+^ T-cells, such as KLRG1, and CD57 [9]. These TEX markers also play prognostic roles in other cancers. LAG3 is considered as the third checkpoint inhibitor in metastatic melanoma [15]. PD1 and CTLA4 blockade demonstrates impressive tumor control in patients with melanoma [16]. Expression of TIM3 or PD1 negatively correlates with survival of patients with hepatocellular carcinoma [17]. CD8^+^CD57^+^ T-cells in non-small cell lung cancer displayed poor response to PD1 blockade compared with CD8^+^CD57^-^ counterparts [18]. The reinvigoration of CD8TEX indicates great therapeutic potential and may be a breakthrough in improving ICB efficacy [19].

In this study, we extracted TEX-related genes using the single-cell RNA sequencing (ScRNA-seq) dataset from the Tumor Immune Single-Cell Hub (TISCH) database. These genes were then combined with differentially expressed genes (DEGs) between PACA tumors and control tissues from the Cancer Genome Atlas (TCGA) and the Genotype-Tissue Expression (GTEx) databases to construct the TEX prognostic risk model. We identified that the TEX risk model could predict the outcomes and TIME status of patients with PACA. Our analysis demonstrated that the TEX-related gene signature is an independent and promising prognostic model for PACA.

## 2. Materials and Methods

### 2.1. Data Source and Clinical Information

Integrated gene expression and clinical information of PACA patients and control subjects in TCGA and GTEx databases were downloaded from Xena (https://xena.ucsc.edu/, accessed on 1 January 2024), an online platform for public, multi-omic and clinical/phenotype data. ICGC-PACA-CA and ICGC-PAAD-US PACA cohorts were retrieved from the International Cancer Genome Consortium (ICGC, https://dcc.icgc.org/, accessed on 1 January 2024). The GSE71729 dataset was downloaded from the Gene Expression Omnibus (GEO, https://www.ncbi.nlm.nih.gov/geo/, accessed on 1 January 2024). All ScRNA-seq datasets were downloaded from TISCH, a scRNA-seq database including 190 datasets focusing on TME. Adjusted *p*-value < 0.05 and |log2 FC| > 2 were used as cutoff values for filtering differentially expressed genes (DEGs) in bulk sequencing datasets. Adjusted *p*-value < 0.05 and |log2 FC| > 0.3 were used as cutoff values for filtering DEGs in scRNA-seq datasets. DEseq2 (v4.41.12), edgeR (v3.40.2), and limma (v3.54.2) R packages were used for DEGs analysis. Benjamini–Hochberg (BH) was used to obtain the adjusted *p*-value in DEG analysis using DEseq2, edgeR, and limma.

### 2.2. Construction of T-Cell Exhaustion Signature

DEGs from TCGA-PAAD and GTEx were intersected with DEGs of CD8TEX from TISCH to screen CD8TEX-related DEGs. Univariate Cox regression and Kaplan–Meier survival analysis of overall survival (OS) were performed with *p*-value cutoff of 0.05 to filter OS correlated TEX-related DEGs using the survival (v3.3.1) R package. LASSO regression was followed by stepwise variable selection process to obtain the best candidate for Cox’s proportional hazards model using the glmnet (v4.1-7) and My.stepwise (v0.1.0) R packages. The final risk model was constructed by multivariate Cox regression analysis using the survival (v3.3.1) R package, and the forest plot was drawn using the forestplot (v3.1.1) R package. The PACA patients were divided into high- and low-risk subgroups with the cutoff of median risk scores.

### 2.3. Validation of T-Cell Exhaustion-Related Risk Model

In the training TCGA-PAAD dataset, Kaplan–Meier survival analysis was performed to compare the OS between high- and low-risk groups. Risk score curve, patient distribution, and expression of TEX-related genes in the risk prognostic model were also compared using the tinyarray (v2.2.9) R package. The 1-, 3-, and 5-year survival curves were constructed and the area under the curve (AUC) was determined using the survival (v3.3.1) and survminer (v0.4.9) R packages and plotted with the timeROC (v0.4) R package [20]. Univariate and multivariate Cox regression analyses were performed to identify independent prognostic factors including age, gender, grade, stage, and risk score. A nomogram was drawn to assess risk based on these characteristics using the rms (v6.6-0) R package. Decline curve analysis (DCA) was used to evaluate the clinical applicability of the nomogram via quantifying the improved benefits using the ggDCA (v1.2) R package.

In validation cohorts, including ICGC-PACA-CA (*n* = 186), ICGC-PAAD-US (*n* = 112), and GSE71729 (*n* = 114), KM survival analysis was performed to validate the prognostic prediction potential of the TEX-related gene signature. Risk score curve, patient distribution, and expression of TEX-related genes in the risk prognostic model were also compared using tinyarray (v2.2.9)

### 2.4. Immune Cell Infiltration Estimation

The gene signatures for immune cell type classification and gene signatures for immune function were extracted from a previous study [21]. The immune cell abundance and immune function estimation were performed using the “ssgsea” function with the GSVA R package. Moreover, XCELL, TIMER, QUANTISEQ, and CIBERSORT algorithms were applied to estimate the immune cell infiltration using the immunedeconv (v2.1.3) and CIBERSORT (v0.1.0) R packages [22]. The spearman correlation between the infiltration ratio and risk score was performed using the psych (v2.3.3) R package, and a *p*-value of 0.05 was used as threshold.

### 2.5. Immune Subtype Analysis

Tumors could be classified into six immune subtypes [23], named C1 to C6. C1 (wound healing) is characterized by high proliferation rate. C2 (IFN-γ dominant) had a strong CD8 signal and the highest M1/M2 macrophage polarization. C3 (inflammatory) had low somatic copy number alterations and tumor cell proliferation. C4 (lymphocyte depleted) displayed a more prominent macrophage signature and high M2 response. C5 (immunologically quiet), was dominated by M2 macrophages and exhibited the highest macrophage responses but the lowest lymphocyte responses. C6 (TGF-β dominant) had the highest TGF-β signature. The TCGA-PAAD patients were classified using the ImmuneSubtypeClassifier (v0.1.0) R package.

### 2.6. Drug Response Prediction

The Genomics of Drug Sensitivity in Cancer 2 (GDSC2, https://www.cancerrxgene.org/, accessed on 1 January 2024) database consists of 805 types of cells and their response to 198 antitumor drugs. The cell line expression and half-maximal inhibitory concentration (IC50) data were downloaded GDSC2. The drug response of the TCGA-PAAD patients to these compounds was predicted using the oncoPredict (v0.2) R package [24]. The correlation between drug response and risk score was calculated using the psych (v2.3.3) R package and plotted with ggstatsplot (v0.0.6) and ggpubr (v0.6.0) R packages.

### 2.7. Immunohistochemistry Staining

The surgical PACA tumor and adjacent tissues were briefly deparaffinized and rehydrated, followed by incubation with primary antibodies overnight (anti-SPOCK2: 11725-1-AP; anti-MT1X: 7172-1-AP; anti-LIPH: 16602-1-AP) from Proteintech (https://ptgcn.com/products/, accessed on 1 January 2024). These sections were rinsed with PBS, incubated with corresponding secondary antibodies at 37 °C.

### 2.8. Pan-Cancer scRNA-Seq Analysis of CD8TEX-Related Genes

The expression of TEX-related genes was explored and plotted in the PAAD-CRA001160 scRNA-seq dataset from the TISCH database (http://tisch.comp-genomics.org/home/, accessed on 1 January 2024). A pan-cancer expression of SPOCK2 across a variety of cell types was analyzed and plotted in heatmap and violin plots.

### 2.9. Statistical Analysis

All statistical analysis was carried out utilizing R software (v4.2.3). Comparison of mean values between different groups was performed using Student’s *t*-test. The correlation analysis was assessed using Pearson correlation analysis. The log-rank test was used in the Kaplan–Meier survival analysis. A *p*-value of the adjusted *p*-value < 0.05 considered statistically significant.

## 3. Results

### 3.1. Identification of TEX-Related Gene Signature in PACA

**Figure 1:**
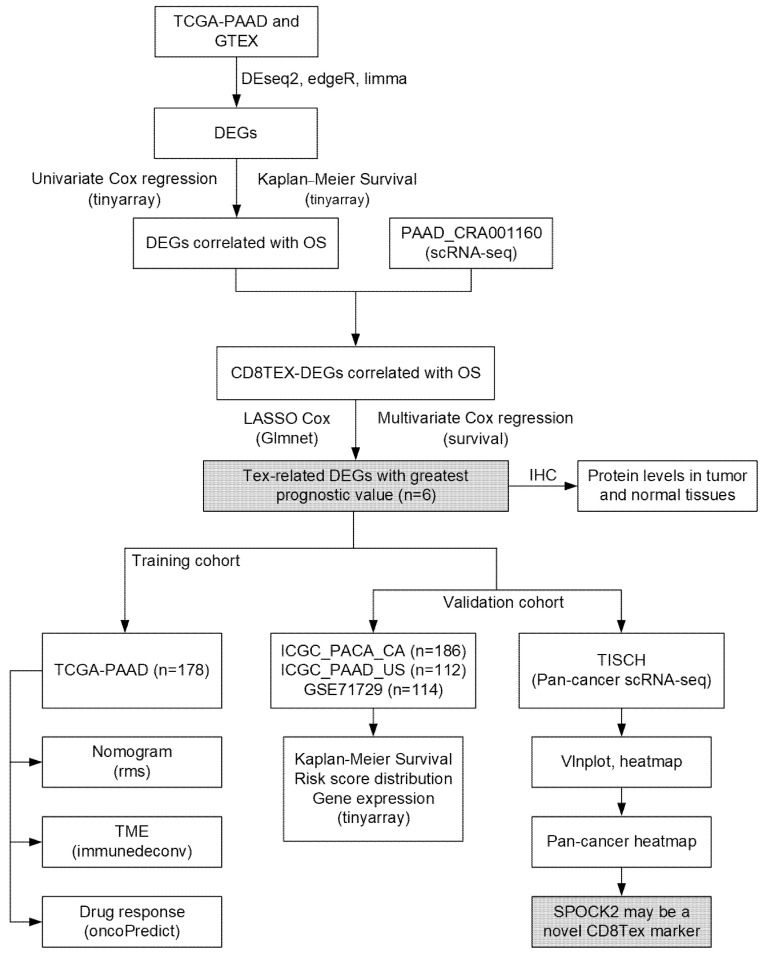
The general workflow of the current study. Common DEGs were identified by intersecting the results from three methods (DESeq2, edgeR, and limma), and were further screened by univariate Cox regression and Kaplan–Meier survival analysis to select DEGs with prognostic potential, which were intersected with CD8TEX. Finally, the risk model was constructed by multivariate Cox regression using genes obtained after LASSO regression. The risk model was validated in several datasets. scRNA-seq: single-cell RNA sequencing; DEGs: differently expressed genes; OS: overall survival; LASSO: least absolute shrinkage and selection operator; IHC: immunohistochemistry; TME: tumor microenvironment.

The general workflow of the current study is depicted in Figure 1.

### 3.2. Differential Expression Analysis

The TCGA-GTEx cohort included 179 tumor samples from the TCGA database. Four adjacent tissue samples from TCGA and 167 normal human pancreatic tissue samples from GTEx were used as controls. A robust disease-specific model with fewer genes is more suitable for clinical translation; therefore, only the common DEGs that were identified as significant across all three methods (DESeq2, edgeR, and limma R packages) were considered. Additionally, 3156 DEGs were identified across all three programs, including 1985 upregulated and 1171 downregulated genes (Table S1).

### 3.3. Prognostic Potential Analysis of DEGs

We further explored the prognostic potential of these DEGs using univariate Cox regression and Kaplan–Meier survival analyses. The surv_cox function from the tinyarray (v2.2.9) R package was used to calculate the Cox *p*-value and hazard ratio (HR) of DEGs in univariate Cox regression with a *p*-value cutoff of 0.05. A total of 1150 DEGs with prognostic potential were identified (Table S2). The surv_KM function from the tinyarray (v2.2.9) R package was used to calculate the log_rank test *p*-value for DEGs in the Kaplan–Meier survival analysis with a *p*-value cutoff of 0.05. Subsequently, 681 DEGs with prognostic potential were identified (Table S3).

### 3.4. Identification of TEX-Related Gene Signature in PACA

**Figure 2:**
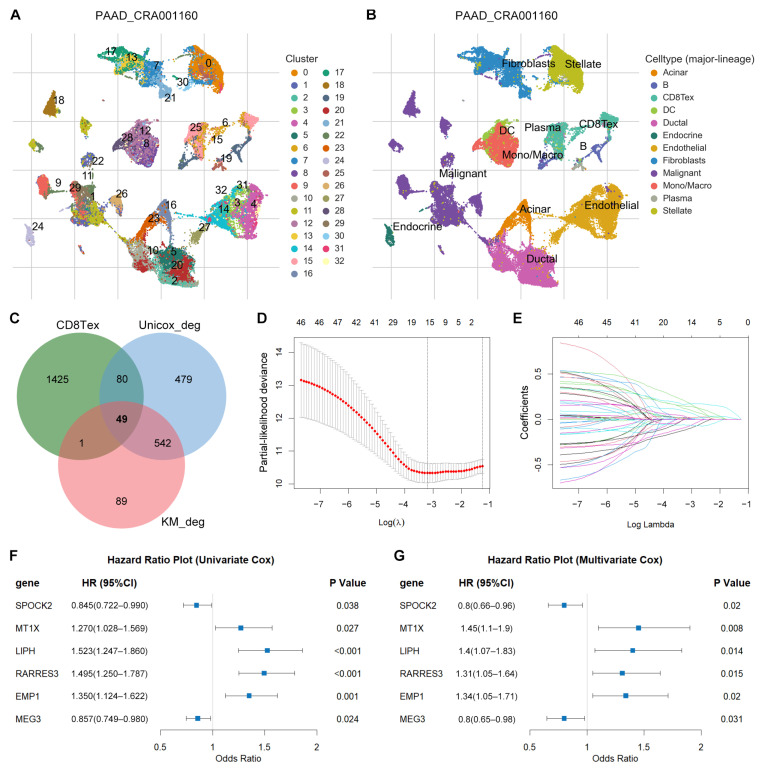
Identification of TEX-related gene signature in PACA. (**A**) The UMAP plot in PAAD-CRA001160 scRNA-seq dataset from TISCH2 database. These cells were distributed in 33 clusters. (**B**) The UMAP plot presented by cell type. The CD8TEX mainly consisted of cells from cluster 6 and cluster 15. (**C**) Intersection among CD8TEX-related genes and DEGs selected by three methods (DESeq2, edgeR, and limma R packages) with prognostic values in both univariate Cox regression and Kaplan–Meier survival analysis. This intersected Venn plot was generated using the jvenn online tool, which is an interactive Venn plot viewer (https://jvenn.toulouse.inra.fr/app/example.html, accessed on 1 January 2024). (**D**,**E**) The LASSO coefficient profiles generated by 49 prognostic TEX-related DEGs using the survival (v3.3.1) and glmnet (v4.1-7) R packages. The log(λ).min was used to select the optimal gene sets, and a total of 14 TEX-related DEGs were selected for stepwise variable selection process to obtain the best candidate for Cox’s proportional hazards model. Six TEX-related DEGs were finally utilized by stepwise variable selection process with the My.stepwise (v0.1.0) R package. (**F**,**G**) Univariate and multivariate Cox regression analyses of the six TEX-related DEGs confirmed the significant association with OS in PACA patients from TCGA using survival (v3.3.1) and survminer (v0.4.9) R packages. The forest plot was drawn with the forestplot (v3.1.1) R package.

The TISCH2 database is a scRNA-seq database that includes 190 datasets focusing on the TME. Model-based AnalysEs of Transcriptome and RegulOme (MAESTRO) were utilized in TISCH2 to perform quality control, normalization, clustering, and cell type annotation for each collected dataset [25]. In total, 1555 genes associated with CD8TEX were obtained from the PAAD-CRA001160 scRNA-seq dataset in the TISCH2 database (Figure 2A,B; Table S4). The intersecting genes among the CD8TEX genes and DEGs identified with prognostic potential using univariate Cox regression and Kaplan–Meier survival analysis were categorized as TEX-related DEGs. A total of 49 TEX-related DEGs were identified (Figure 2C, Table S5).

LASSO regression, followed by stepwise variable selection, was used to obtain the best candidate for the Cox proportional hazards model (Figure 2D,E). The final TEX risk model was constructed using multivariate Cox regression analysis including six genes (*SPOCK2*, *MT1X*, *LIPH*, *RARRES3*, *EMP1*, and *MEG3*). Both univariate and multivariate Cox regression analysis confirmed the correlation between these risk genes and OS in patients with PACA (Figure 2F,G).

### 3.5. Validation of TEX-Related Gene Signature in PACA

**Figure 3:**
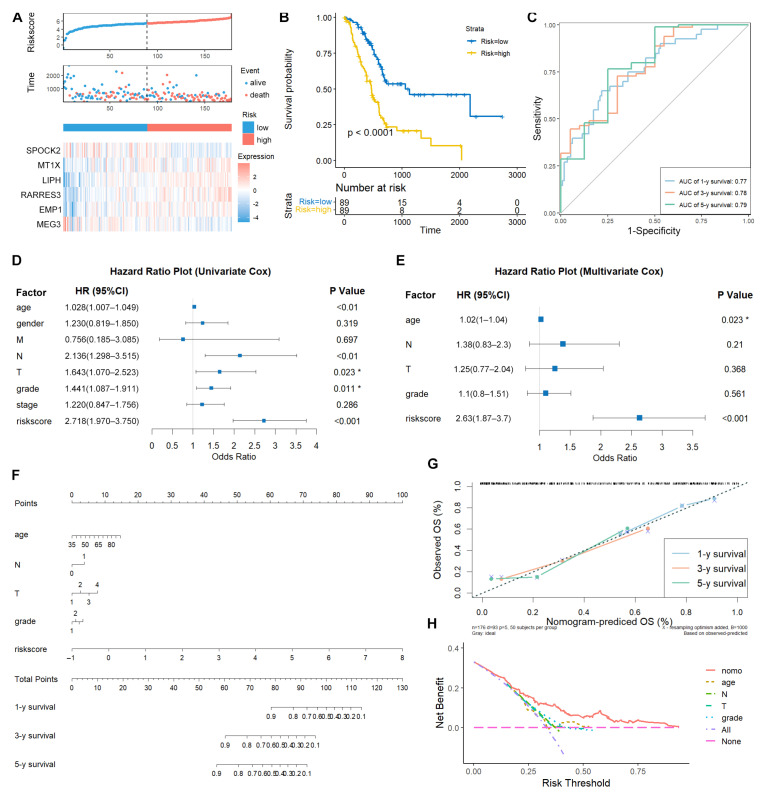
Validation of TEX-related gene signature in PACA. (**A**) Risk score and patient distribution and expression of risk genes in the TCGA-PAAD cohort were shown using the risk_plot function from the tinyarray (v2.2.9) R package. More alive patients were found in the low-risk group. (**B**) Kaplan–Meier curve of the high- and low-risk patients drawn using survival (v3.3.1) and survminer (v0.4.9) R packages. (**C**) The ROC curve for 1-, 3-, and 5-year survival in TCGA-PAAD patients drawn using the timeROC (v0.4) R package. (**D**,**E**) Univariate and multivariate Cox regression analyses of the risk score and clinical factors demonstrated the risk score was an independent prognostic factor for PACA patients. The risk score served as a risk factor for PACA patients. The survival (v3.3.1) and survminer (v0.4.9) R packages were used for analysis, and the forest plot was drawn with the forestplot (v3.1.1) R package. (**F**) The nomogram plot was constructed based on clinical factors and the risk score. (**G**) Calibration plot of the nomogram in TCGA-PAAD patients. Both nomogram and calibration plots were drawn using the rms (v6.6-0) R package. (**H**) DCA curve of the nomogram, age, N stage, T stage, and grade for TCGA-PAAD patients. The DCA curve was drawn using the ggDCA (v1.2) R package. * represents *p*-value < 0.05.

The risk score was calculated using the following formula: riskscore = −0.2248802 × SPOCK2 + 0.3692181 × MT1X + 0.3364109 × LIPH + 0.2723827 × RARRES3 + 0.2901199 × EMP1 − 0.2231695 × MEG3. Patients from TCGA-PAAD were used as the training dataset and divided into high- and low-risk groups based on the median risk score. SPOCK2 and MEG3 were downregulated, whereas MT1X, LIPH, and RARRES3 were upregulated in the high-risk group (Figure 3A). Kaplan–Meier survival analysis demonstrated that low-risk patients had longer survival times than high-risk patients (Figure 3B). Moreover, the ROC curve based on the TEX-related signature demonstrated AUC values of 0.77, 0.78, and 0.79 for 1-, 3-, and 5-year time points, respectively (Figure 3C).

We further explored whether this risk model could act as in independent prognostic variable in patients with PACA. Both univariate and multivariate Cox regression analyses confirmed that the risk score and age were significantly correlated with OS (Figure 3D,E). To make this risk model clinically useful, a nomogram was constructed to predict 1-, 3-, and 5-year survival in TCGA-PAAD patients. As depicted in the nomogram, the risk score model had the highest weight among all included factors, including age, N stage, T stage, and grade (Figure 3F). Calibration curves showed consensus between the nomogram-predicted and actual survival rates (Figure 3G). DCA curves demonstrated that the nomogram performed better than the other clinical factors in predicting the 1-, 3-, and 5-year OS (Figure 3H).

**Figure 4:**
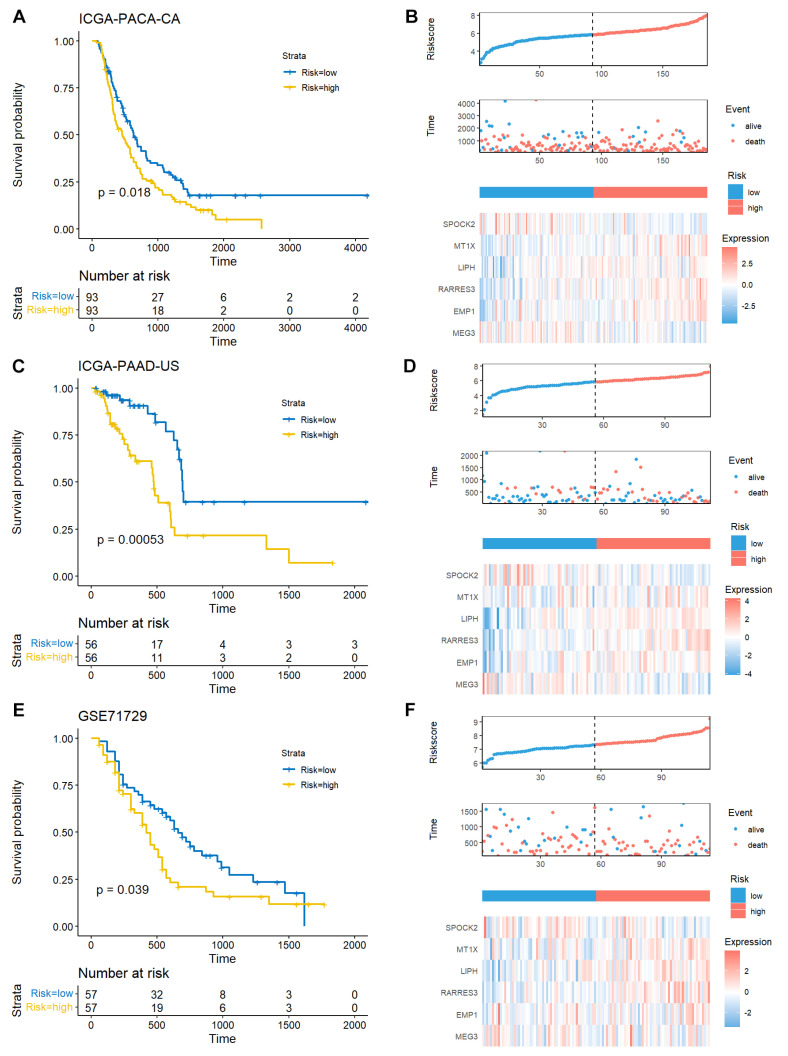
External validation of TEX-related gene signature in PACA. Kaplan–Meier curve of the high- and low-risk patients, risk score and patient distribution, and expression of risk genes in ICGC-PACA-CA (**A**,**B**), ICGC-PAAD-US (**C**,**D**), and GSE71729 (**E**,**F**) cohorts. The Kaplan–Meier curves were drawn using survival (v3.3.1) and survminer (v0.4.9) R packages. The risk score and patient distribution were drawn using the risk_plot function from the tinyarray (v2.2.9) R package. More alive patients were found in the low-risk group across all three datasets.

Three external validation datasets (ICGC-PACA-CA, ICGC-PAAD-US, and GSE71729, *n* = 412) were used to validate the prognostic potential of the TEX-related signature. A total of 186 (34 alive and 152 dead), 112 (68 alive and 44 dead), and 114 (34 alive and 80 dead) patients with survival times longer than 30 days were included in the ICGC-PACA-CA, ICGC-PAAD-US, and GSE71729 datasets, respectively. Consistent with the results of the TCGA-PAAD training dataset, low-risk patients had prolonged survival times, and the expression patterns of the risk genes were similar to those in the TCGA cohort (Figure 4A–F). Collectively, these results from different cohorts suggest that the TEX-related gene signature may be an independent predictive indicator for patients with PACA.

### 3.6. Immune Characteristics of High- and Low-Risk Groups

**Figure 5:**
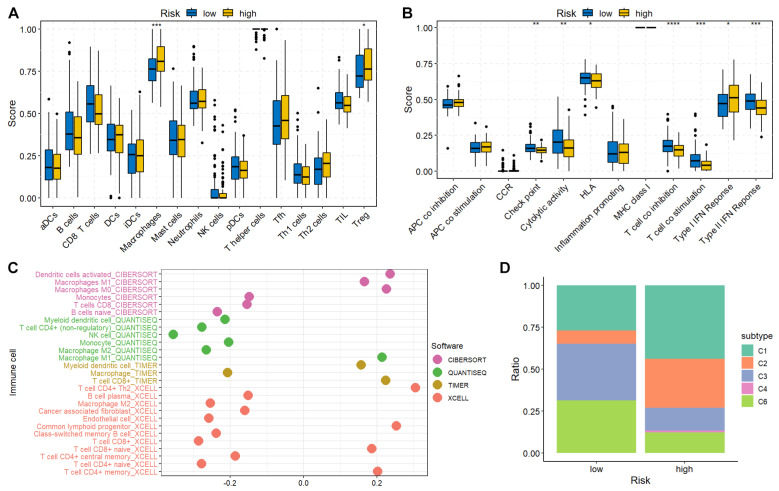
Immune characteristics of high- and low-risk groups. (**A**) The immune cell infiltration scores between high- and low-risk groups. Macrophages and regulatory T-cells (Treg) were enriched in the high-risk group. (**B**) The immune function scores between high- and low-risk groups. The gene set enrichment was performed using the GSVA (v1.46.0) R package, and the box plots were drawn using the ggpubr (v0.6.0) R package. (**C**) Correlation between immune cell abundance and risk score. Immune cell infiltration was calculated using immunedeconv (v2.1.3) and CIBERSORT (v0.1.0) R packages. (**D**) The distribution of PACA patients in different immune subtypes was analyzed using the ImmuneSubtypeClassifier (v0.1.0) R package and plotted using the ggplot2 R package. * represents *p*-value < 0.05, ** represents *p*-value < 0.01, *** represents *p*-value < 0.001, **** represents *p*-value < 0.0001.

The abundance and function of immune cells were estimated using previously published gene signatures [21]. Immune cell infiltration and immune function differences between the high- and low-risk groups were compared using the Wilcoxon test. High-risk patients tended to have increased numbers of macrophages and regulatory T-cells (Treg) (Figure 5A). Decreased checkpoint, cytolytic activity, human leukocyte antigen, T-cell co-stimulation, and type II interferon response were observed in the high-risk group (Figure 5B), indicating an immunosuppressive TME in high-risk patients. Additionally, the correlation between immune cell infiltration and risk score was investigated utilizing numerous algorithms. The results demonstrated that the risk score was negatively correlated with natural killer (NK) and CD4^+^ T-cells (Figure 5C). Moreover, patients with PACA were further classified into immune subtypes C1-C6. Interestingly, C5 (immunologically quiet) was absent in both groups, indicating the malignant features of PACA. The presence of C4 (lymphocyte depleted) only in the high-risk group, characterized by a prominent macrophage signature and high M2 response, suggested an immunosuppressive status (Figure 5D).

### 3.7. The Correlation between Drug Sensitivity and Risk Score

**Figure 6:**
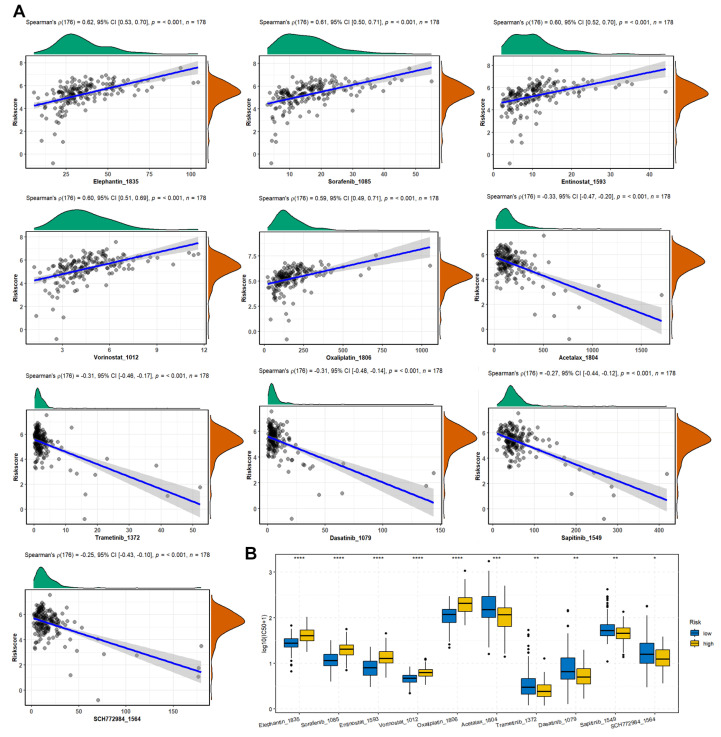
The correlation between drug sensitivity and risk score. (**A**) Spearman correlation between drug IC50 and risk score was analyzed using the corr.test function from the psych (v2.3.3) R package, and the scatter plot was drawn using the ggstatsplot (v0.0.6) R package. (**B**) Differences in drug IC50 between high- and low-risk groups were examined by Wilcoxon test and the box plot was drawn using the ggpubr (v0.6.0) R package. * represents *p*-value < 0.05, ** represents *p*-value < 0.01, *** represents *p*-value < 0.001, **** represents *p*-value < 0.0001.

The predicted IC50 values for TCGA-PAAD patients were obtained using the OncoPredict R package based on the GDSC2 database. Spearman correlation analysis demonstrated that the risk scores were positively correlated with elephantin, sorafenib, entinostat, vorinostat, oxaliplatin, and negatively correlated with acetalax, trametinib, dasatinib, sapitinib, and SCH772984 (Figure 6A). Moreover, the positively correlated drugs displayed significantly decreased IC50 values in the low-risk group compared to those in the high-risk group (Figure 6B).

### 3.8. Expression Validation of Risk Genes in PACA Tissues

**Figure 7:**
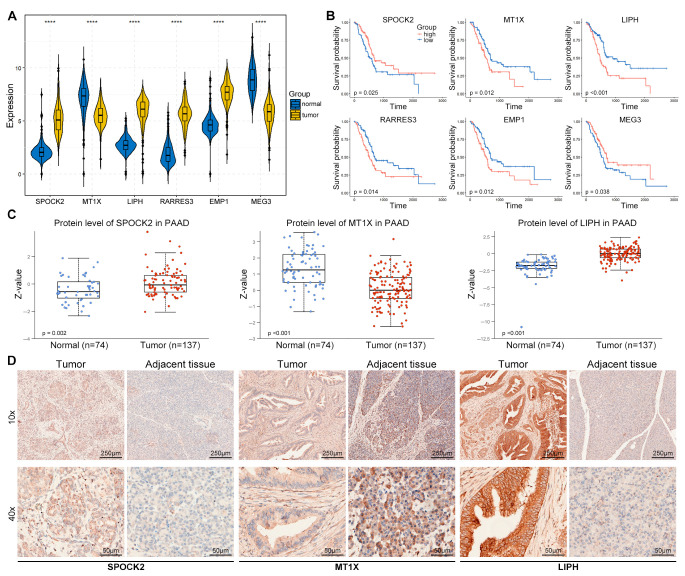
Expression validation of risk genes in PACA tissues. (**A**) The mRNA levels of risk genes in the TCGA-GTEx cohort. The differences were examined by Wilcoxon test and the box plot was drawn using the ggpubr (v0.6.0) R package. (**B**) The Kaplan–Meier curve of the high- and low-risk patients based on the expression of risk genes in TCGA cohort drawn using survival (v3.3.1) and survminer (v0.4.9) R packages. (**C**) The protein levels of SPOCK2, MT1X, and LIPH in PACA from the CPTAC database (https://ualcan.path.uab.edu/index.html, accessed on 1 January 2024). (**D**) IHC staining of SPOCK2, MT1X, and LIPH in PACA tumor and adjacent tissues from untreated male PACA patients (*n* = 3). **** represents *p*-value < 0.0001.

We further explored the expression of risk genes in the PACA tissues. The messenger RNA (mRNA) levels of these genes in the TCGA-GTEx cohort displayed that SPOCK2, LIPH, RARRES3, and EMP1 were significantly higher in tumor samples than in control tissues (Figure 7A). The Kaplan–Meier curve of the high- and low-risk patients based on the expression of risk genes in the TCGA cohort demonstrated that SPOCK2 and MEG3 were positively correlated with the OS of patients with PACA, while MT1X, LIPH, RARRES3, and EMP1 were negatively correlated with the OS of patients with PACA (Figure 7B). The protein expression of several tumors from the Clinical Proteomic Tumor Analysis Consortium (CPTAC) was analyzed using the University of Alabama at Birmingham CANcer data analysis Portal (UALCAN, https://ualcan.path.uab.edu/index.html, accessed on 1 January 2024) [26]. The results from the CPTAC database confirmed that the protein levels of SPOCK2 and LIPH were remarkably increased in PACA tumors compared to those in control tissues, whereas the protein level of MT1X was significantly decreased in PACA tumors (Figure 7C). In addition, our IHC staining demonstrated results consistent with those of the CPTAC database; SPOCK2 and LIPH were increased, whereas MT1X was decreased in untreated patients with PACA (Figure 7D).

### 3.9. SPOCK2 May Be a Novel CD8TEX Cellular Marker

**Figure 8:**
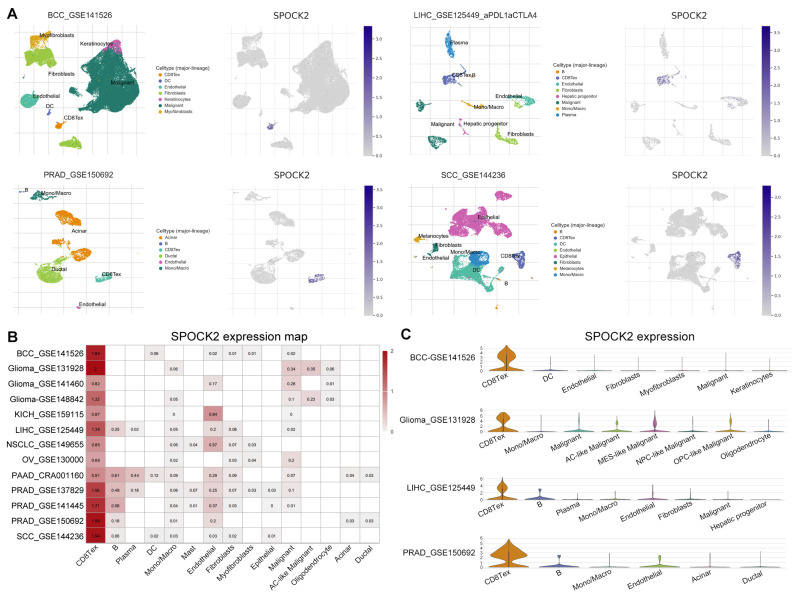
SPOCK2 may be a novel CD8TEX cellular marker. (**A**) Feature plots of SPOCK2 expression in several tumors, and SPOCK2 was specifically and highly expressed in CD8TEX cells. (**B**) Heatmap of SPOCK2 expression across numerous tumors. (**C**) Violin plots of SPOCK2 expression in BCC, glioma, LIHC, and PRAD. BCC: basal cell carcinoma; LIHC: liver hepatocellular carcinoma; PRAD: prostate adenocarcinoma; SCC: squamous cell carcinoma; KICH: kidney chromophobe; NSCLC: non-small cell lung carcinoma; OV: ovarian serous cystadenocarcinoma.

The understanding of tumor heterogeneity at the single-cell level has been promoted by scRNA-seq [27]. We explored the expression of risk genes in TME using the PAAD-CRA001160 dataset from the TISCH database. Interestingly, SPOCK2 was expressed almost exclusively in CD8TEX cells. We further explored the expression of SPOCK2 in some tumors, including basal cell carcinoma (BCC), liver hepatocellular carcinoma (LIHC), prostate adenocarcinoma (PRAD), and squamous cell carcinoma (SCC). Similarly, SPOCK2 was highly expressed in CD8TEX cells of BCC, LIHC, PRAD, and SCC (Figure 8A). A heatmap was generated to illustrate the expression levels of SPOCK2 across numerous tumors, including glioma, kidney chromophobe (KICH), non-small cell lung carcinoma (NSCLC), and ovarian serous cystadenocarcinoma (OV). The results revealed that SPOCK2 was specifically and highly expressed in CD8TEX cells compared to its expression in B-cells, dendritic cells, monocytes, macrophages, fibroblasts, and malignant cells (Figure 8B,C). Collectively, these scRNA-seq results of numerous tumors strongly suggest that SPOCK2 may serve as a novel CD8TEX cellular marker.

## 4. Discussion

The lack of effective treatment makes PACA one of the most lethal malignancies, although some advances have been made in surgical intervention, adjuvant therapy, and chemotherapy [28]. Immunotherapy provides revolutionary strategies for the treatment of cancer and has rejuvenated the tumor immunology field [29], which is considered as the primary treatment for several tumors [30]. Several types of immunotherapy, including ICIs, T-cell transfer therapy, monoclonal antibodies, treatment vaccines, and immune system modulators, have achieved durable clinical responses [31]. However, PACA remains almost unresponsive to immunotherapy due to low mutational loads, desmoplastic dense stroma, low number of tumor neoantigens, and “cold” TIME [32]. Recent advancements in scRNA-seq have facilitated a systematic interrogation of the PACA TME, offering novel insights into the annotation of precise cellular compositions in the PACA TME [33]. TEX-related genes can be extracted from CD8TEX cells from the scRNA-seq database. Additionally, TEX-based gene signatures have been widely used to predict prognostic, molecular, and immune features, as well as therapeutic responses in several tumors, including LIHC, lung adenocarcinoma, and NSCLC [34,35,36,37]. In this study, we identified and validated a TEX-related signature for predicting the prognosis and immune response in PACA using integrated analysis of scRNA-seq and bulk RNA sequencing data.

Cancer therapy has been revolutionized over the past decade by ICB therapy. The initial paradigm was that ICB targeting anti-PD-1/PD-L1 directly reversed exhaustion within PD-1^hi^CD8^+^ T-cells in the TME locally. However, recent studies have determined that ICB activity is not locally restricted to the TME and is a result of the trafficking of ICB-permissive, stem-like precursor CD8^+^ T-cells outside the tumor to the TME [38]. The CXCR3 ligands, CXCL9 and CXCL10, are critical mediators of intratumoral CD8^+^ T-cell trafficking [39]. Furthermore, CCL5–CCR5 interactions played essential roles in the initial recruitment of CD8^+^ T-cells and are remarkably correlated with the response to anti-PD-1/PD-L1 ICB [40].

These data revealed that ICB irradiation elicited the expansion and mobilization of stem-like CD8^+^ T-cells within tumor-draining lymph nodes. These findings suggest that ICB activity depends on communication between the tumor, blood, and lymphoid immune compartments. Moreover, liquid biopsy samples may be used to identify clinical biomarkers and facilitate the early prediction of ICB treatment response. Two independent studies demonstrated that ICB-induced immunomodulation of CD8^+^ T-cells in the blood of responding patients with melanoma, but not in those who did not respond to treatment [41,42].

CD8TEX cell-related genes were extracted from the PACA scRNA-seq dataset in the TISCH database and merged with DEGs with prognostic potential in the TCGA-GTEx cohort. Subsequently, univariate Cox regression, LASSO regression, and stepwise multivariate Cox regression analyses were performed to construct a final TEX-related risk model consisting of the six genes. In the training TCGA cohort, we discovered that low-risk patients had increased survival times and that the TEX-related risk model was an independent prognostic indicator with a more accurate prediction of clinical outcomes than other clinical factors such as age, grade, and stage. The prognostic potential of this risk model was validated in three external cohorts (ICGC-PACA-CA, ICGC-PAAD-US, and GSE71729), indicating its robustness.

The TIME was also investigated to characterize its organization and immune function and to develop new treatments for PACA. High-risk patients exhibit an immunosuppressive TME characterized by increased infiltration of Treg cells and decreased antigen presentation, cytolytic activity, and checkpoint immunological functions. Moreover, Treg cells, characterized by the expression of FOXP3, are an immunosuppressive subset of CD4^+^ T-cells that play essential roles in maintaining self-tolerance and hampering antitumor immune responses [43]. Moreover, the risk score is negatively correlated with NK and CD4^+^ T-cells, which are essential components of innate and adaptive immune cells. In addition, high-risk patients have unique immune subtypes. The lymphocyte depleted C4 subtype, characterized by a high M2 response, was observed only in the high-risk group.

It should be noted that PACA TME is much more complex than just the lymphocytes themselves. Other cell types and their complex interactions within the TME are essential for the understanding of tumor immune evasion and selection of therapeutic strategies, such as macrophages, fibroblasts, and myeloid-derived suppressor cells. The complex interactions of cancer cells with stromal cells and extracellular matrix (ECM) components within the TME is critical to stimulate the tumor heterogeneity and to induce tumor immune evasion. The tumor cell hijacking of non-malignant cells stimulates stromal cells to acquire new phenotypes promoting cancer cell progression and metastasis. The dynamic and bidirectional interaction of cancer cells with their TME components includes cell–cell contacts, cell-free contacts, and mediators enabling these interactions. Mediators are secreted soluble molecules or vesicles responsible for the transfer of genetic information between communicating cells. Numerous strategies, including disrupting the tumor–stromal cell interaction by anti-angiogenic therapy, cancer-associated fibroblast depletion, immune reprogramming, exosome/circulating tumor cell targeting, and hyaluronic acid depletion, have been used to combat these tumors [44]. Further study of the functions and interactions of various cells and non-cellular components like ECM within the TME will aid in demonstrating the complex mechanisms governing these interactions and inducing tumor immune evasion and may uncover novel therapeutic targets.

Recent studies have reported positive results for immunotherapy via epigenetic transcriptional control. Li et al. reported that KDM3A regulated the expression of epidermal growth factor receptors to suppress antitumor immunity in PACA. Genetic ablation of KDM3A reduces the infiltration of cancer-associated fibroblasts, promotes T-cell infiltration, and sensitizes tumors to combination immunotherapy [45]. We identified that risk scores were positively correlated with several drugs. Moreover, the positively correlated drugs demonstrated significantly decreased IC50 values in the low-risk group compared to those in the high-risk group, which may provide combination immunotherapy choice for further investigations. The IC50 of sorafenib, a kinase inhibitor, was positively correlated with the risk score in patients with PACA. A recent meta-analysis of randomized trials of patients with advanced hepatocellular carcinoma (HCC) revealed that the survival outcomes of sorafenib have improved over time in advanced HCC [46]. The IC50 of the histone deacetylase inhibitor entinostat positively correlated with the risk score in patients with PACA. Entinostat converts immune-resistant PACA into checkpoint-responsive tumors by reprogramming tumor-infiltrating myeloid-derived suppressor cells [47]. These data may have essential implications for PACA clinical trial design.

In addition to the mRNA expression of risk genes, we validated the protein levels of these risk genes in both the proteomic database and our clinical specimens. Consistent with the gene expression changes in PACA, SPOCK2 and LIPH increased, whereas MT1X decreased in PACA. Finally, we investigated the expression pattern of these risk genes within the TME of PACA and discovered that SPOCK2 was specifically and highly expressed in CD8TEX cells in numerous tumors, including BCC, glioma, KICH, LIHC, NSCLC, OV, PACA, PRAD, and SCC. Collectively, these scRNA-seq results at the single-cell level suggest that SPOCK2 may serve as a novel CD8TEX cellular marker.

SPOCK2 is a secreted protein that plays essential roles in OV carcinogenesis, endometrial cancer, PRAD, and LUAD [48]. Both mRNA and protein levels of SPOCK2 were decreased in LUAD. High SPOCK2 levels correlated with favorable outcome in LUAD, which is consistent with our result in PACA. SPOCK2 is strongly correlated with the GTPases of the immunity-associated protein family involved in the regulation of lymphocyte survival and homeostasis. Moreover, SPOCK2 was positively associated with the infiltration levels of CD8^+^ T-cells, and played an important role in regulating tumor-infiltrating immune cells in LUAD [48]. The expression and prognostic role of SPOCK2 in PACA and other tumors, such as OV, are both similar and different. SPOCK2 expression increased in both PACA and OV. Kaplan–Meier analysis demonstrated that patients with PACA with high SPOCK2 mRNA expression had better OS than those with low expression, whereas patients with high-grade serous ovarian cancer with high SPOCK2 mRNA expression had worse OS than the OS of those with low expression. These results indicated the functional heterogeneity of SPOCK2 in different tumors. Further investigation of the regulatory roles of SPOCK2 in the development and differentiation, expression of immune checkpoint and senescence genes, metabolism, and function of CD8^+^ T-cells may help understand the underling mechanism of SPOCK2 in PACA. Metallothioneins (MTs) are cysteine-rich, low molecular weight proteins that play a crucial role in the carcinogenesis of diverse malignancies. MT1 includes 13 members, including MT1A, MT1B, and MT1X. In the current study, we discovered that MT1X expression was negatively correlated with the OS of patients with PACA. Similarly, Masiulionytė B et al. demonstrated that high MT1X expression was associated with shortened survival in patients with glioma [49]. RARRES3 is a tumor suppressor in several cancers. Loss of RARRES3 is a key driver of lung metastasis in estrogen receptor-positive breast cancer, and the activation of RARRES3 is considered a useful therapeutic strategy for bladder cancer [50,51]. MEG3 serves as a tumor suppressor in breast cancer by influencing on the expression of miR-182 and miR-29 [52]. These genes displayed differential expression between patients with PACA and the control participants and were significantly correlated with the survival of patients with PACA. A predictive risk model with great prognostic potential was constructed using these genes, although they played distinctly opposite functions.

In conclusion, we established and validated a TEX-related prognostic signature in patients with PACA. Moreover, our study suggests that SPOCK2 is a novel marker of exhausted CD8^+^ T-cells. This study provides deep insights into TEX in PACA.

## Data Availability

The TCGA and GTEx integrated data were downloaded from Xena (https://xenabrowser.net/datapages/?cohort=TCGA%20TARGET%20GTEx&removeHub=https%3A%2F%2Fxena.treehouse.gi.ucsc.edu%3A443, accessed on 1 January 2024). ICGC-PACA-CA was downloaded from ICGC (https://dcc.icgc.org/projects/PACA-CA, accessed on 1 January 2024). ICGC-PAAD-US was downloaded from ICGC (https://dcc.icgc.org/projects/PAAD-US, accessed on 1 January 2024). The GSE71729 dataset was downloaded from GEO (https://www.ncbi.nlm.nih.gov/geo/query/acc.cgi?acc=GSE71729, accessed on 1 January 2024). Other data and results are displayed in the manuscript and Supplementary Data.

## Supplementary Materials

The following supporting information can be downloaded at: https://www.mdpi.com/article/10.3390/diagnostics14060667/s1, Table S1: DEGs between tumor and normal from TCGA-GTEX dataset using DEseq2, edgeR, and limma R packages; Table S2: Univariate Cox analysis of overall survival (OS) using Differentially expressed genes from TCGA-GTEx dataset; Table S3: Kaplan-Meier analysis using Differentially expressed genes from TCGA-GTEx dataset; Table S4: CD8Tex genes from PAAD_CRA001160 scRNA-seq dataset; Table S5: TEX-related DEGs.

## Abbreviation

PACA	pancreatic cancer
PAAD	pancreatic adenocarcinoma
TIME	tumor immune microenvironment
TME	tumor microenvironment
ICB	immune checkpoint blockade
TEX	T-cell exhaustion
ScRNA-seq	single-cell RNA sequencing
TISCH	Tumor Immune Single-Cell Hub
TCGA	Cancer Genome Atlas
GTEx	Genotype-Tissue Expression
ICGC	International Cancer Genome Consortium
GEO	Gene Expression Omnibus
DEGs	differentially expressed genes
OS	overall survival
BCC	basal cell carcinoma
LIHC	liver hepatocellular carcinoma
PRAD	prostate adenocarcinoma
SCC	Squamous cell carcinoma
KICH	kidney chromophobe
NSCLC	non-small cell lung carcinoma
OV	ovarian serous cystadenocarcinoma
